# *In silico* modeling for *ex vivo* placenta perfusion of nicotine

**DOI:** 10.3389/fphar.2024.1275467

**Published:** 2024-04-12

**Authors:** Harvey Ho, Shengjie Zhang, Ken Kurosawa, Botao Jiang, Koji Chiba

**Affiliations:** ^1^ Auckland Bioengineering Institute, The University of Auckland, Auckland, New Zealand; ^2^ Department of Clinical Pharmacology, Janssen Pharmaceutical K.K., Tokyo, Japan; ^3^ Department of Urology, Xianning Central Hospital, The First Affiliated Hospital Of Hubei University Of Science And Technology, Xianning, Hubei Province, China; ^4^ Laboratory of Clinical Pharmacology, Yokohama University of Pharmacy, Yokohama, Kanagawa, Japan

**Keywords:** nicotine, placenta, *Ex vivo* perfusion, In silico model, transplacental

## Abstract

Nicotine readily crosses the placenta to reach fetuses. However, membrane transporters, e.g., organic cation transporters (OCTs) play a role in the clearance of nicotine from the fetal to the maternal side, and this is rarely investigated clinically. In this work, we use an *in silico* model to simulate an *ex vivo* placenta perfusion experiment, which is the gold standard for measuring the transplacental permeability of compounds, including nicotine. The model consists of a system of seven ordinary differential equations (ODEs), where each equation represents the nicotine concentration in compartments that emulate the *ex vivo* experiment setup. The transport role of OCTs is simulated bi-directionally at the placenta’s basal membrane (the fetal side). We show that the model can not only reproduce the actual *ex vivo* experiment results, but also predict the likely maternal and fetal nicotine concentrations when the OCT transporters are inhibited, which leads to a ∼12% increase in fetal nicotine concentration after 2 hours of OCT modulated nicotine perfusion. In conclusion, a first *in silico* model is proposed in this paper that can be used to simulate some subtle features of trans-placental properties of nicotine.

## 1 Introduction

Maternal smoking is a public health concern as it leads to many adverse effects on fetuses, including changes in fetal heart rate and breathing movements, congenital malformation, and stillbirth, to name a few (Lambers and Clark, 1996; Humphrey et al., 2016). Nicotine replacement therapy (NRT), where nicotine patches, gums, or medicine are administered to pregnant women, aims to assist the smoking cessation efforts of pregnant women (Benowitz and Dempsey, 2004). It has been shown that pregnant women have an accelerated metabolism of nicotine and cotinine, the major metabolite of nicotine (Benowitz and Dempsey, 2004; Benowitz et al., 2009). As a compound of low molecular weight and high lipid solubility, nicotine readily crosses the placenta (Pastrakuljic et al., 1998; Taghavi et al., 2018). Therefore, it seems that the placenta plays a little role as a barrier for nicotine from the maternal to fetal side (m → f) (Pastrakuljic et al., 1998). However, the extraction of nicotine from the fetal to the maternal side (f → m) is more complicated, as the process is modulated by organic cation transporters (OCTs) (Lin et al., 2022), and possibly other transporters, e.g., P-gp as well (McColl et al., 2023). In human beings, OCT3 is the only OCT isoform expressed at placental basal membrane vesicles (Sata et al., 2005). Using a rat model, Lin et al. (2022) investigated the transplacental pharmacokinetics of nicotine and cotinine modulated by OCT. Indeed, it has been reported that the nicotine concentration on the fetal side is ∼15% higher than on the maternal side (Lambers and Clark, 1996). Efforts to alleviate the impact of nicotine on fetuses, therefore, need to address the problem of the clearance of nicotine f → m, and understand the underlying mechanisms.

Unfortunately, clinical investigations on this topic are very rare due to the difficulties of collecting blood samples from fetuses. Previous studies, e.g., of Luck et al. (1985), measured nicotine and cotinine concentration at different stages of gestation from amniotic fluid, umbilical vein serum/maternal vein serum, and placenta tissue. The study, however, only indirectly investigates the transplacental properties of nicotine. In comparison, *ex vivo* placenta perfusion experiments provide an excellent setup to measure the transplacental permeability of drugs (Kopecky et al., 1999). In such an experiment, a cotyledon of a freshly harvested placenta is cannulated from both maternal and fetal sides, and connected to reservoirs. Drug concentrations are measured from the reservoirs at both sides, and the permeability of the drug is thus determined. *Ex vivo* placental experiments have been performed for a number of drugs, including metformin (Kovo et al., 2008), morphine (Kopecky et al., 1999). *Ex vivo* placental experiments for nicotine are rare. One study was performed in 1998 (Pastrakuljic et al., 1998), where the authors determined nicotine and cotinine transplacental rates. They found that cotinine was not detectable in both maternal and fetal reservoirs, implying a very limited metabolism of nicotine in the human placenta.

However, the *ex vivo* study of (Pastrakuljic et al., 1998) has certain limitations. Firstly, the harvested placenta only reflects the placental physiology at the time of childbirth, yet the metabolism of nicotine changes greatly during pregnancy (Lambers and Clark, 1996). Secondly, the study did not investigate the modulation role of OCT transporters on the transplacental perfusion of nicotine. It was shown that inhibition of OCT transporters by corticosterone substantially increased nicotine concentration in fetal circulation (Lin et al., 2022). This drug-drug interaction scenario has not been investigated clinically, to our knowledge. This study aims to build a first *in silico* model which can be utilized to study the transplacental properties of nicotine.

## 2 Methods

### 2.1 Mathematical model and data source

In the *ex vivo* experiments of (Pastrakuljic et al., 1998), placentas were collected and sent to laboratories within 30 min of the delivery of infants. One isolated cotyledon of the placenta was then cannulated from the maternal and fetal sides, and maternal and fetal circulations were established separately in either “closed” or “open” circuit design. This study only simulates the closed circuit, as it is more similar to the actual physiological scenario (Kurosawa et al., 2020). Practically, a cell culture media perfusate filled the maternal and fetal reservoirs, where the maternal perfusate initially contained 40 ng/mL of nicotine. This nicotine concentration is typical in plasma after cigarette smoking in adults (Russell et al., 1975). With flow rates of approximately 13–15 mL/min on the maternal side and 3–4 mL/min on the fetal side, reservoir samples (L mL) were obtained every 5 min for the first half hour, and subsequently every half hour. The data were used to verify our computational model.

**FIGURE 1:**
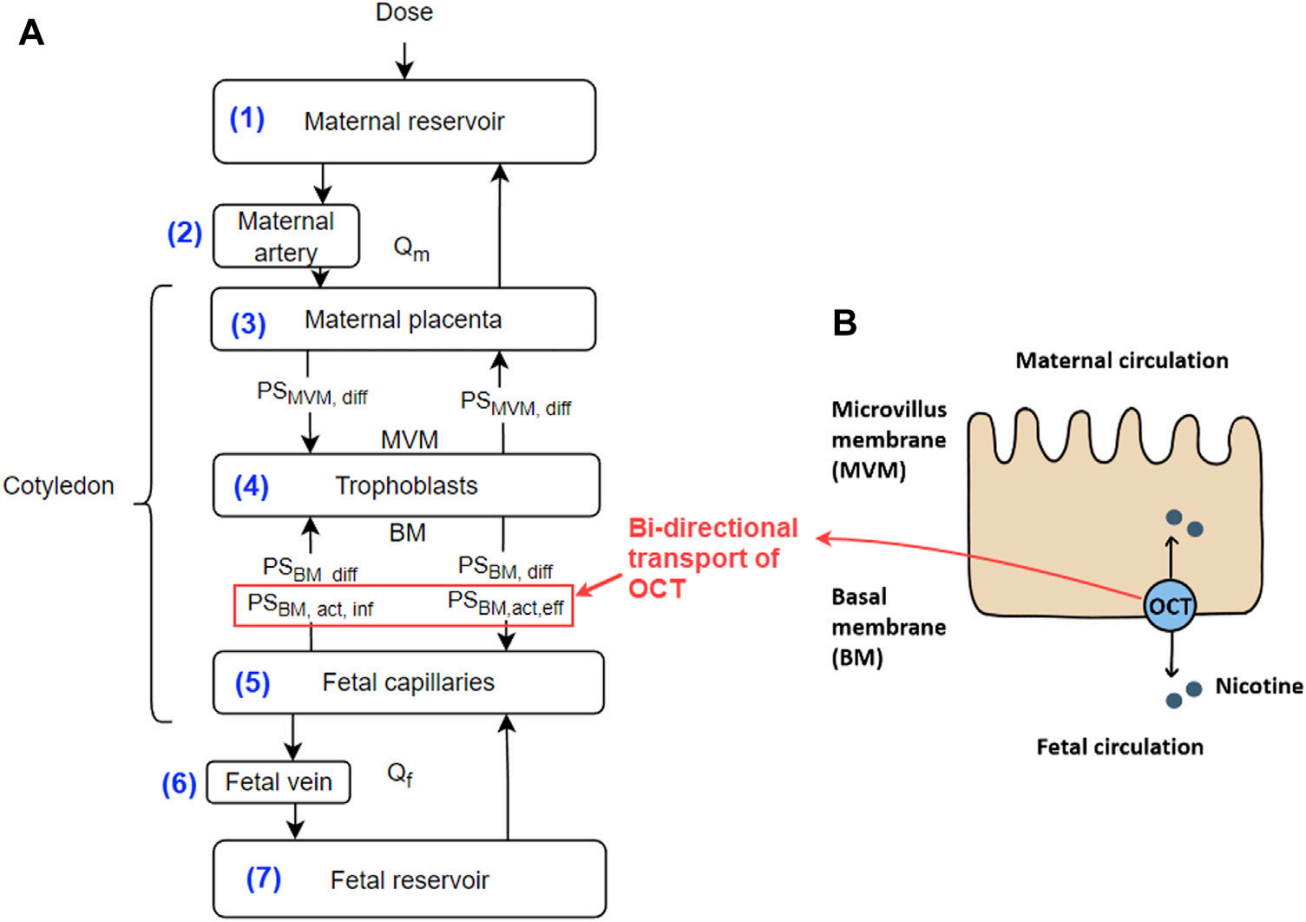
**(A)** Conceptual representation of the *ex vivo* human cotyledon perfusion system. The numbers for the compartments correspond to their respective equation numbers. Explanations of the parameters are provided in the text; **(B)** Diagram showing the OCT meditated transport of nicotine across the placenta. Note, that the OCT transporters play a bi-directional transport role in our model. Abbreviations: BM - basal membrane; MVM - microvillus membrane; *Q*_*m*_ and *Q*_*f*_ - flow rate at the maternal and fetal side, respectively.

Based on our previous study of *ex vivo* perfusion for morphine (Ho et al., 2022), the nicotine model was constructed that contains seven ordinary differential equations (ODEs). The conceptual representation of the model is shown in Figure 1A, where each of the equations represents a corresponding compartment according to the *ex-vivo* human cotyledon perfusion system (Ho et al., 2022). The ODE system is listed below.

$$\frac{dC_{mr}}{dt}=\frac{Q_{m}\times \left(C_{mp}-C_{mr}\right)}{V_{mr}}$$ (1)

$$\frac{dC_{ma}}{dt}=\frac{Q_{m}\times \left(C_{mr}-C_{ma}\right)}{V_{ma}}$$ (2)

$$\frac{dC_{mp}}{dt}=\frac{Q_{m}\times \left(C_{ma}-C_{mp}\right)+PS_{\mathrm{MV M,diff}}\times C_{t}-PS_{\mathrm{MV M,diff}}\times C_{mp}}{V_{mp}}$$ (3)

$$\frac{dC_{t}}{dt}=\frac{PS_{\mathrm{MV M,diff}}\times C_{mp}+\left(PS_{BM,act,inf}+PS_{BM,diff}\right)\times C_{fc}}{V_{t}}$$ (4)

$$\begin{array}{ll} & \;\times \frac{-\left(PS_{\mathrm{MV M,diff}}+PS_{BM,diff}+PS_{BM,act,eff}\right)\times C_{t}}{V_{t}}\\ \frac{dC_{fc}}{dt} & =\frac{Q_{f}\times \left(C_{fr}-C_{fc}\right)+\left(PS_{BM,diff}+PS_{BM,act,eff}\right)\times C_{t}}{V_{fc}} \end{array}$$ (5)

$$\begin{array}{ll} & \times \frac{-\left(PS_{BM,act,inf}+PS_{BM,diff}\right)\times C_{fc}}{V_{fc}}\\ \frac{dC_{fv}}{dt} & =\frac{Q_{f}\times \left(C_{fc}-C_{fv}\right)}{V_{fv}} \end{array}$$ (6)

$$\frac{dC_{fr}}{dt}=\frac{Q_{f}\times \left(C_{fv}-C_{fr}\right)}{V_{fr}}$$ (7)

where the concentrations, blood flow, and distribution volume of each compartment *i* are represented by *C*_*i*_, *Q*_*i*_ and *V*_*i*_, respectively. *i* refers to the corresponding compartments, i.e., maternal circulation m), fetal circulation f), maternal reservoir (mp), maternal artery (ma), maternal placenta (mp), trophoblast t), fetal capillaries (fc), fetal vein (fv) or fetal reservoir (fr) (Figure 1A). Note, nicotine crosses the placenta in both passive diffusion and active transport pathways. The coefficients of passive diffusion on the microvillus membrane (MVM, the maternal side) and basal membrane (BM, the fetal side) are represented by *PS*_*MVM, diff*_ and *PS*_*BM*, *diff*_, respectively. Active influx activity through OCT transporters was found on the BM side of the placenta and is represented by *PS*_*BM*,*act*,*inf*_ and *PS*_*BM*,*act*,*eff*_ to reflect the bi-directional transport role of OCTs (Figure 1B).

The model was implemented in MATLAB in Version R2021b (MathWorks, USA). The ODE solver was ODE45. Table 1 shows the nominal value of the model’s parameters.

**TABLE 1:** Physiological and pharmacokinetic parameters for the nicotine model.

Parameters	Notes	Value	Monolix fitting	References
Q_*m*_	Flow rate of the perfusate within the maternal circulation	15 mL/min		Pastrakuljic et al. (1998)
Q_*f*_	Flow rate of the perfusate in the fetal circulation	3 mL/min		Pastrakuljic et al. (1998)
V_*mr*_	Volume of maternal reservoir	250 mL		Pastrakuljic et al. (1998)
V_*fr*_	Volume of fetal reservoir	150 mL		Pastrakuljic et al. (1998)
V_*mp*_	Volume of maternal placenta	74.7 mL		Ho et al. (2022)
V_*ma*_	Volume of maternal artery	0.15 mL		Ho et al. (2022)
V_*fc*_	Volume of fetal capillary	1.34 mL		Ho et al. (2022)
V_*t*_	Volume of trophoblast	2.68 mL		Ho et al. (2022)
V_*fv*_	Volume of fetal vein	0.15 mL		Ho et al. (2022)
PS_*MVM, diff*_	Diffusion rate on MVM membrane	15.12 mL/min	91.87	Fitted
PS_*BM*, *diff*_	Diffusion rate on BM membrane	12.13 mL/min	5.04	Fitted
PS_*BM*,*act*,*inf*_	Active influx transport on the BM	0.9 mL/min	0.17	Fitted
PS_*BM*,*act*,*eff*_	Active efflux transport on the BM	1.2 mL/min	0.0000044	Fitted

^a^ MVM, microvillus membrane; BM, basal membrane.

### 2.2 Nonlinear mixed effects modeling

Nonlinear mixed effect modelling is a computational method for population pharmacokinetics, and can also be used to derive parameter values to fit a mathematical model to experimental data (Chan et al., 2011). To further investigate the effect of OCT-modulated active transport, the model was also implemented in Monolix Suite 2023R1 (Lixoft, France). We fixed all the physiological parameters *V*_*i*_ and *Q*_*i*_, while applying random effects to the four parameters *PS*_*MVM, diff*_, *PS*_*BM*, *diff*_, *PS*_*BM*,*act*,*eff*_, and *PS*_*BM*,*act*,*inf*_. We used the proportional error model suggested by Monolix, and performed with correlation between all four parameters.

## 3 Results

### 3.1 Simulation without OCT inhibition

**FIGURE 2:**
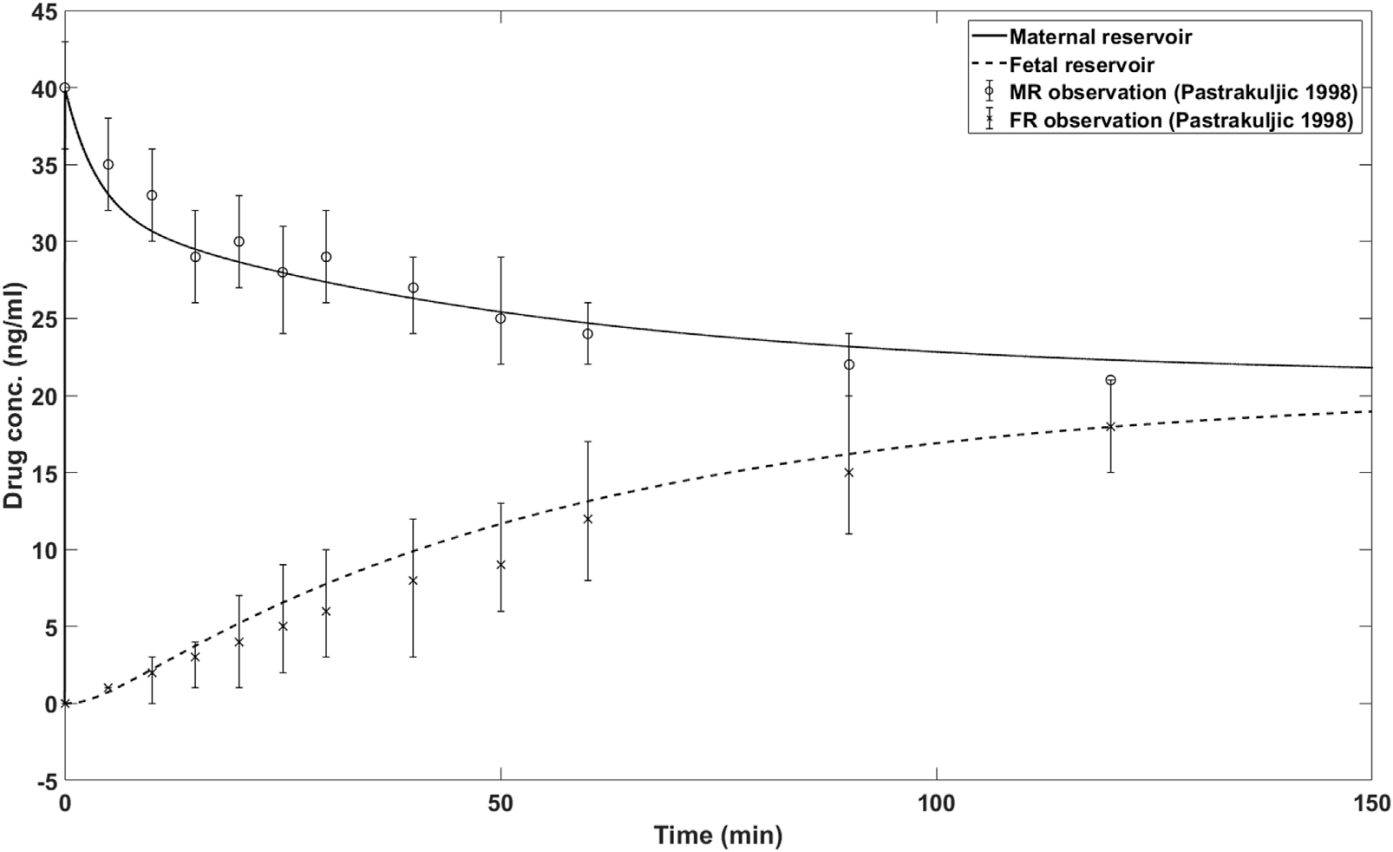
*In silico* model simulations and reported nicotine concentrations for maternal and fetal reservoir. MR: maternal reservoir; FR: fetal reservoir.

The simulation results of the *in silico* model with presented parameter values in Table 1 are shown in Figure 2. Also shown are the *ex vivo* transplacental data reported in (Pastrakuljic et al., 1998). As can be seen, the simulation results closely match the actual *ex vivo* data.

### 3.2 Simulation with OCT inhibition

**FIGURE 3:**
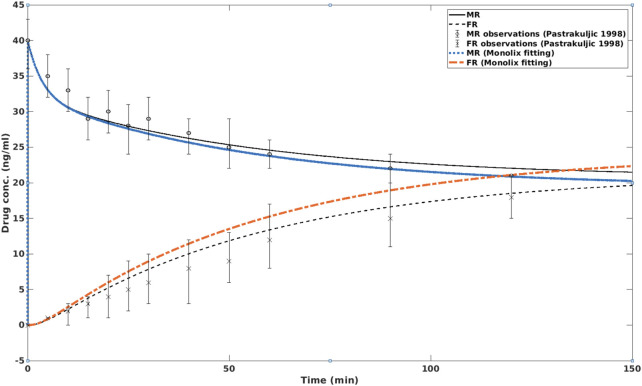
*In silico* simulation of nicotine concentrations in MR and FR with and without the presence of OCT inhibition. In the former case, the fetal nicotine concentration is higher than in the latter case, i.e., without OCT inhibition. After about 2 h, the nicotine concentration on the fetal side is higher than on the maternal side. MR: maternal reservoir; FR: fetal reservoir.

Although the OCT-modulated transplacental perfusion of nicotine has been reported for the rat placenta (Lin et al., 2022), it has not been reported for the human placenta, to our knowledge. Nevertheless, an *in silico* simulation for *ex vivo* transplacental perfusion of human placenta was made by adjusting the four parameters *PS*_*MVM, diff*_, *PS*_*BM*, *diff*_, *PS*_*BM*,*act*,*eff*_ and *PS*_*BM*,*act*,*inf*_, to fit virtual results that are likely to happen in human placenta. The results are shown in Figure 3.

In this simulation, it can be seen that OCT inhibition enhanced the nicotine level in the fetal circulation. After approximately 2 hours, the fetal nicotine concentration becomes higher than that at the maternal side. At 150 min, the difference between fetal and maternal nicotine concentrations reaches 11.9%. This is consistent with the clinical study of (Luck et al., 1985), which reported that the ratio of umbilical vein serum vs. maternal vein serum was 1.12 ± 0.30 (or approximately 12% higher) at birth.

## 4 Discussion

In the majority of developed countries, the maternal smoking rate is about 10% (Dessì et al., 2018). This rate may be considerably higher in certain subpopulations. For example, the pregnancy smoking rate among the Maori population, the indigenous people in New Zealand, reached 32% (Humphrey et al., 2016). To reduce the adverse effects of maternal smoking on fetuses, it is important to understand the mechanism of nicotine clearance from fetal circulation. In this study, we used an *in silico* model to simulate the *ex vivo* transplacental nicotine perfusion results of a previous study (Pastrakuljic et al., 1998), where nicotine quickly crossed the placenta to reach the fetal circulation. In addition, we simulated the scenario where the return of nicotine f → m was facilitated by a combined passive perfusion and active transport via OCT. Different from some other placental transport mechanisms, e.g., clearance of morphine from the fetal circulation, where the efflux transporter P-gp is expressed at the basolateral or maternal side (Ho et al., 2022), OCT is expressed at the apical or fetal side.

When the transplacental pathway modulated by OCT is inhibited, the nicotine concentration in fetal circulation is significantly higher than without inhibition, as shown in the rat model (Lin et al., 2022), and our simulation results (Figure 3). Therefore, it may be speculated that clearance of nicotine from fetal circulation could be improved through induction of the OCT pathway. The challenge is that OCT is a bi-directional transporter, i.e., it facilitates both influx and efflux of nicotine at the basal membrane. Their interactions could be a key to nicotine clearance from fetuses. Clinical reports on the nicotine concentration changes due to OCT modulation are few. However, the mechanism provides valuable insights into reducing fetal exposure to nicotine in NRT therapies.

From a computational perspective, the *in silico* model has a similar topological structure (Figure 1A) to other *ex vivo* transplacental models, e.g., of Kurosawa et al. (2020) and Ho et al. (2022). The differences lie in whether the *ex vivo* experiment is designed as “open” or “closed” circulation at the maternal and fetal sides. In addition, since each drug has its pharmacological characteristics (e.g., lipid solubility), and may be a substrate to different transporters, the subtleties of the model, e.g., perfusion or transport of the drug at the cotyledon membrane, linear or nonlinear efflux/influx across the placental barrier, and whether the drug is metabolised within the placenta, etc., depends on each drug. Therefore, the model needs to be tuned and adjusted accordingly for these subtleties. In terms of parameter fitting, we used two software packages, i.e., Matlab and Monolix. It seems that Monolix has superior performance in NONMEM and associated parameter fitting procedures. The convergence of the proportional error model was robust in Monolix. However, it may be argued that the fitted four parameters are not a unique set of parameters. There may have other sets of parameters that could yield similar fitting results. Further *in vitro* or *ex vivo* experiments will need to be designed to verify parameters associated with nicotine permeation at the MVM and BM membranes.

Concerning the *ex vivo* placental perfusion experiment itself, it is worth noting that there may have some large variations between the *in vivo* placental perfusion rate and the established *ex vivo* flow rate, e.g., 3–4 mL/min at the fetal side for a single isolated cotyledon (Pastrakuljic et al., 1998). For a term placenta, the mean umbilical venous flow rate is about 400 mL/min (Barbera et al., 1999). Since a human placenta contains about 15–20 cotyledons (Calis et al., 2022), the *in vivo* perfusion rate at the fetal side per cotyledon is therefore approximately 20 mL/min. Therefore, the blood flow rate and shear stress in an *in vivo* environment could be higher than that in an *ex vivo* setup. Higher *in vivo* perfusion rates in the placenta can lead to a faster transfer rate of nicotine m → f, but this hypothesis requires future investigations to verify.

There are other limitations to the current study. Firstly, we did not consider the transplacental transfer of cotinine in the model. The reason was that the *ex vivo* study was not able to detect cotinine in the maternal and fetal reservoirs (Pastrakuljic et al., 1998). This implies that the placenta itself has limited metabolic capabilities for transforming nicotine into cotinine. However, as the most important metabolite of nicotine, it is important to investigate the transplacental properties of cotinine separately. Secondly, in the experiments of (Pastrakuljic et al., 1998), only placentas from mothers with no history of cigarette smoking, and chronic drug therapy exposure were harvested for perfusion experiments. It may be argued that the placentas of smoking mothers have different responses to nicotine exposure. Nevertheless, the model presented in this study can be further tuned to simulate these scenarios when new data are available.

## 5 Conclusion

We presented an *in silico* model for *ex vivo* placenta perfusion of nicotine. The model not only reproduces the nicotine concentrations of an actual *ex vivo* placenta model, but further simulates the likely placenta perfusion with OCT inhibition. The model is valuable for the research of maternal and fetal health.

## Data Availability

The original contributions presented in the study are included in the article/Supplementary material, further inquiries can be directed to the corresponding authors.

## References

[B1] Barbera A. Galan H. L. Ferrazzi E. Rigano S. Józwik M. Battaglia F. C. (1999). Relationship of umbilical vein blood flow to growth parameters in the human fetus. Am. J. obstetrics Gynecol. 181, 174–179. 10.1016/s0002-9378(99)70456-4 10411816

[B2] Benowitz N. L. Dempsey D. A. (2004). Pharmacotherapy for smoking cessation during pregnancy. Nicotine Tob. Res. 6, S189–S202. 10.1080/14622200410001669169 15203821

[B3] Benowitz N. L. Hukkanen J. Jacob III P. (2009). Nicotine chemistry, metabolism, kinetics and biomarkers. Nicotine Psychopharmacol., 29–60. 10.1007/978-3-540-69248-5_2 PMC2953858 19184645

[B4] Calis P. Vojtech L. Hladik F. Gravett M. G. (2022). A review of *ex vivo* placental perfusion models: an underutilized but promising method to study maternal-fetal interactions. J. Maternal-Fetal Neonatal Med. 35, 8823–8835. 10.1080/14767058.2021.2005565 PMC9126998 34818981

[B5] Chan P. L. Jacqmin P. Lavielle M. McFadyen L. Weatherley B. (2011). The use of the saem algorithm in monolix software for estimation of population pharmacokinetic-pharmacodynamic-viral dynamics parameters of maraviroc in asymptomatic HIV subjects. J. Pharmacokinet. pharmacodynamics 38, 41–61. 10.1007/s10928-010-9175-z PMC3020311 21088872

[B6] Dessì A. Corona L. Pintus R. Fanos V. (2018). Exposure to tobacco smoke and low birth weight: from epidemiology to metabolomics. Expert Rev. proteomics 15, 647–656. 10.1080/14789450.2018.1505508 30052087

[B7] Ho H. Zhang S. Kurosawa K. Chiba K. (2022). *In silico* modeling for *ex vivo* placental transfer of morphine. J. Clin. Pharmacol. 62, 140–146. 10.1002/jcph.2105 36106779 PMC9543479

[B8] Humphrey G. Rossen F. Greenaway N. Bullen C. (2016). Parental smoking during pregnancy: findings from the growing up in New Zealand cohort 27657160

[B9] Kopecky E. A. Simone C. Knie B. Koren G. (1999). Transfer of morphine across the human placenta and its interaction with naloxone. Life Sci. 65, 2359–2371. 10.1016/s0024-3205(99)00503-2 10597891

[B10] Kovo M. Haroutiunian S. Feldman N. Hoffman A. Glezerman M. (2008). Determination of metformin transfer across the human placenta using a dually perfused *ex vivo* placental cotyledon model. Eur. J. Obstetrics Gynecol. Reproductive Biol. 136, 29–33. 10.1016/j.ejogrb.2007.01.013 17350747

[B11] Kurosawa K. Chiba K. Noguchi S. Nishimura T. Tomi M. (2020). Development of a pharmacokinetic model of transplacental transfer of metformin to predict *in vivo* fetal exposure. Drug Metabolism Dispos. 48, 1293–1302. 10.1124/dmd.120.000127 33051249

[B12] Lambers D. S. Clark K. E. (1996). The maternal and fetal physiologic effects of nicotine. Seminars perinatology (Elsevier) 20, 115–126. 10.1016/s0146-0005(96)80079-6 8857697

[B13] Lin I.-H. Yang L. Dalley J. W. Tsai T.-H. (2022). Trans-placental transfer of nicotine: modulation by organic cation transporters. Biomed. Pharmacother. 145, 112489. 10.1016/j.biopha.2021.112489 34915670

[B14] Luck W. Nau H. Hansen R. Steldinger R. (1985). Extent of nicotine and cotinine transfer to the human fetus, placenta and amniotic fluid of smoking mothers. Dev. Pharmacol. Ther. 8, 384–395. 10.1159/000457063 4075937

[B15] McColl E. R. Kwok J. Benowitz N. L. Patten C. A. Hughes C. A. Koller K. R. (2023). The effect of tobacco use on the expression of placental transporters in Alaska native women. Clin. Pharmacol. Ther. 113, 634–642. 10.1002/cpt.2737 36053152 PMC10234256

[B16] Pastrakuljic A. Schwartz R. Simone C. Derewlany L. O. Knie B. Koren G. (1998). Transplacental transfer and biotransformation studies of nicotine in the human placental cotyledon perfused *in vitro* . Life Sci. 63, 2333–2342. 10.1016/s0024-3205(98)00522-0 9877223

[B17] Russell M. A. Wilson C. Patel U. A. Feyerabend C. Cole P. V. (1975). Plasma nicotine levels after smoking cigarettes with high, medium, and low nicotine yields. Br. Med. J. 2, 414–416. 10.1136/bmj.2.5968.414 1168517 PMC1681802

[B18] Sata R. Ohtani H. Tsujimoto M. Murakami H. Koyabu N. Nakamura T. (2005). Functional analysis of organic cation transporter 3 expressed in human placenta. J. Pharmacol. Exp. Ther. 315, 888–895. 10.1124/jpet.105.086827 16081676

[B19] Taghavi T. Arger C. A. Heil S. H. Higgins S. T. Tyndale R. F. (2018). Longitudinal influence of pregnancy on nicotine metabolic pathways. J. Pharmacol. Exp. Ther. 364, 238–245. 10.1124/jpet.117.245126 29158210 PMC5774213

